# Comparing the Impact of COVID-19 on Vaccinated and Unvaccinated Patients Affected by Myasthenia Gravis

**DOI:** 10.3390/life13041064

**Published:** 2023-04-21

**Authors:** Elena Scarsi, Sara Massucco, Pilar M. Ferraro, Arianna Cella, Stefano G. Grisanti, Andrea Assini, Alessandro Beronio, Fabio Della Cava, Chiara Gemelli, Fabio Bandini, Carlo Serrati, Massimo Del Sette, Angelo Schenone, Luana Benedetti, Valeria Prada, Marina Grandis

**Affiliations:** 1 Department of Neurosciences, Rehabilitation, Ophthalmology, Genetic and Maternal and Infantile Sciences (DINOGMI), University of Genoa, Largo P. Daneo 3, 16132 Genova, Italy; 2 Neurology Unit, IRCCS Ospedale Policlinico San Martino, Largo R. Benzi 10, 16132 Genova, Italy; 3 Neurology Unit, Galliera Hospital, Via Mura Delle Cappuccine 14, 16128 Genova, Italy; 4 Department of Neurology, Sant’Andrea Civic Hospital, Via V. Veneto 197, 19121 La Spezia, Italy; 5 Department of Neurology, Imperia Hospital, Via Sant’Agata 57, 18100 Imperia, Italy; 6 Department of Neurology, Villa Scassi Hospital, ASL 3 Genovese, Corso O. Scassi 1, 16149 Genova, Italy

**Keywords:** myasthenia gravis, COVID-19, SARS-CoV-2, coronavirus, vaccine

## Abstract

We evaluated 13 patients affected by myasthenia gravis (MG) who had coronavirus disease 2019 (COVID-19) before vaccination and 14 myasthenic patients who contracted severe acute respiratory syndrome-coronavirus-2 (SARS-CoV-2) infection after vaccination to evaluate factors related to different COVID-19 outcomes. We compared the two groups’ previous stability of MG and the severity of SARS-CoV-2 infection. Vaccinated and non-vaccinated patients were comparable in terms of severity of the previous MG course (mean maximum myasthenia gravis Foundation of America–MGFA–Class III) and during SARS-CoV-2 infection (mean MGFA Class II). In non-vaccinated patients, the hospitalization and severe course percentages were 61.5%, while the mortality reached 30.8%. The hospitalization, severe course, and mortality percentages in vaccinated patients were 7.1%. In deceased, non-vaccinated patients, greater myasthenia severity in the past clinical history, but not at the time of infection, was observed. Similarly, older age at MG onset and at the time of infection correlated with a more severe COVID-19 course in non-vaccinated patients (*p* = 0.03 and *p* = 0.04), but not in the group of vaccinated patients. In summary, our data support a protective role of vaccination in myasthenic patients, even if anti-CD20 therapy might be associated with a poor immune response to vaccines.

## 1. Introduction

Coronavirus disease 2019 (COVID-19) is a respiratory disease caused by severe acute respiratory syndrome-coronavirus-2 (SARS-CoV-2). It was firstly reported in China at the end of 2019 [1] and subsequently spread worldwide.

Patients with myasthenia gravis (MG), an autoimmune disorder affecting the neuromuscular junction, might be particularly vulnerable to SARS-CoV-2 infection, due to weakness of both respiratory and bulbar muscles, long-term immunosuppression, and the well-known role of respiratory infections as a trigger of MG exacerbations and myasthenic crises [2]. Viral infections in general are indeed known to trigger autoimmunity through the augmentation of T cell signaling, causing a pro-inflammatory environment due to a hyper-reactive antiviral immune response and epitope spreading, and they may additionally worsen myasthenic symptoms due to the effects of fever on the function of the neuromuscular junction [3]. Moreover, some drugs used for treating COVID-19 (azithromycin, hydroxychloroquine, and neuromuscular blockers) have been shown to worsen MG muscular weakness [4,5,6].

The analysis of the data from the TriNext COVID-19 Research Network platform, one of the largest international COVID-19 datasets, which includes 380 myasthenic patients who had COVID-19, has revealed that 26.8% of patients required hospitalization, 5.6% experienced MG exacerbation/crisis, and 6.8% died. When COVID-19 myasthenic patients were compared with the entire COVID-19 cohort, MG was found to be associated with a significantly higher risk of hospitalization (odds ratio: 3) and death (odds ratio: 4.3), even after normalization for age and gender [7].

In light of the safety profile of the vaccine among MG patients, Doron and colleagues have recently demonstrated the importance of strongly recommending it to MG patients, especially those with generalized disease forms, due to their significantly increased risk of mortality and severe disease course [8,9,10]. However, doubts remain regarding the effectiveness of the vaccine in patients treated with anti-cluster of differentiation 20 (CD20) monoclonal antibodies, such as rituximab. Previous studies have indeed provided conflicting results, and more importantly, the majority of studies have been focused on other conditions in which these drugs are used, such as multiple sclerosis [11].

In Liguria, a region in Northwestern Italy, we observed several cases of SARS-CoV-2 infection in myasthenic patients with frequent severe outcomes during the first two pandemic waves (between March 2020 and May 2021). In this study, we therefore aimed at investigating which factors in the previous and actual myasthenic clinical history, as well as comorbidities, could be associated with a worse outcome.

Since April 2021, the vaccination campaign for frail patients has begun in Italy, so the additional aim of our study was also to further compare the course of infection between non-vaccinated patients and a different group of myasthenic patients who contracted SARS-CoV-2 infection after vaccination in order to assess the protective effect of the vaccine in myasthenic patients.

## 2. Materials and Methods

In this observational, retrospective study, we included all patients with a generalized myasthenia gravis (gMG) diagnosis followed up in five Ligurian hospitals (both visited by neurologists in person and via telemedicine) who had COVID-19 in the period between March 2020 and May 2021 (corresponding to the first two pandemic waves in Italy) when vaccination was not yet available. We compared these patients with those who contracted the infection after vaccination against SARS-CoV-2. All myasthenic patients included in the study were vaccinated with messenger ribonucleic acid (mRNA) vaccines to avoid the possible increased risk of neuromuscular disease exacerbations associated with live attenuated vaccines.

All patients included in the study had a diagnosis of gMG with antibodies against the acetylcholine receptor (gMG AChR+) or the muscle-specific tyrosine kinase (gMG MuSK+). Patients with a diagnosis of possible MG and with other disorders of the neuromuscular junction were excluded.

The diagnosis of COVID-19 was performed in the presence of a positive polymerase chain reaction (PCR) test for SARS-CoV-2 on a nasopharyngeal swab.

In this study, MG exacerbation was classified as a deterioration of the Myasthenia Gravis Foundation of America (MGFA) clinical classification [12] class that requires rescue therapies such as plasma exchange (PEX) or intravenous immunoglobulin (IVIg) [13], while myasthenic crises were defined as a worsening of muscle weakness resulting in respiratory failure requiring intubation and mechanical ventilation [14].

The authors shared a common Excel database. For each patient, we collected information regarding (1) age, sex, body mass index (BMI), and comorbidities; (2) duration of MG in years, maximum MGFA class reached in clinical history, and MGFA class at the time of SARS-CoV-2 infection; (3) number of myasthenic exacerbations/crises in clinical history and/or during COVID-19; (4) immunosuppressive therapy both in clinical history and at the time of SARS-CoV-2 infection; (5) vaccination against SARS-CoV-2; (6) therapy with monoclonal antibodies against SARS-CoV-2 or antiviral drugs; and (7) severity of COVID-19 course.

The severity of the course of COVID-19 has been classified with a seven-point scale, according to Jakubíková’s paper [15], as follows: 1: asymptomatic COVID-19; 2: isolated symptoms such as anosmia, headache, etc.; 3: mild infection with fatigue, cold, and cough; 4: influenza-like infection without hospital admission; 5: hospitalized patients with proven COVID-19 pneumonia requiring oxygen therapy; 6: severe COVID-19 pneumonia; and 7: death due to COVID-19. Accordingly, we defined severe COVID-19 as a score ≥ 5.

All data were analyzed by using CRAN R version 3.4.1. Differences in the aforementioned variables between vaccinated and non-vaccinated patients, as well as between non-vaccinated deceased and surviving patients were analyzed by using the Mann–Whitney *U*-test and Fisher exact test, as appropriate.

Spearman correlation coefficient analyses were then applied to separately evaluate, in vaccinated and non-vaccinated patients, the associations between clinical features and the severity of the course of COVID-19. The results were considered statistically significant when *p* < 0.05.

## 3. Results

### 3.1. Demographic and MG Features of Vaccinated and Non-Vaccinated Patients

Our study included a population of 13 non-vaccinated patients—6 males (46.2%) and 7 females (53.8%)—with a mean age of 68 years (range 45–86) and with an average duration of MG of 7 years (range 1–28), and 14 myasthenic patients who contracted SARS-CoV-2 infection after vaccination—3 males (21.4%) and 11 females (78.6%)—with a mean age of 63 years (range 22–90) and an average disease duration of 7 years (range 1–23) (Table 1).

All the unvaccinated patients (100%) had a serum positivity of anti-AChR antibodies, while regarding the vaccinated patients, 12 (85.7%) had anti-AchR antibodies, and 2 (14.3%) had anti-MuSK antibodies. None had a diagnosis of seronegative MG (Table 1).

Approximately half of the non-vaccinated patients had a BMI > 25 kg/m^2^ (53.9%), and the same proportion had cardiovascular comorbidities. Only one non-vaccinated patient, who had a history of smoking, had a respiratory comorbidity. Among the vaccinated patients, a significantly smaller percentage of cases (14.3%) had a BMI > 25 kg/m^2^, 2 were affected by type 2 diabetes mellitus, 2 had cardiovascular comorbidities, and 1 had chronic obstructive pulmonary disease (Table 1).

BMI emerged as the only variable which significantly differed between the two groups, with an almost equal frequency of BMI > 25 kg/m^2^ and < 25 kg/m^2^ in non-vaccinated patients and a considerably greater frequency of BMI < 25 kg/m^2^ (85.7%) in vaccinated patients (*p* = 0.04). None of the other demographic and MG features investigated were found to significantly differ between the two groups.

Vaccinated and non-vaccinated patients were indeed comparable in terms of severity of the previous MG course (mean maximum MGFA Class: III) and severity during SARS-CoV-2 infection (mean MGFA Class: II).

A single non-vaccinated patient had a myasthenic crisis in the clinical history, so we considered the number of past myasthenic crises and exacerbations together in our analysis. Based on this classification, five (38.5%) non-vaccinated and eight (57.1%) vaccinated patients were classified as having experienced myasthenic exacerbations in their clinical history (Table 1). 

Two non-vaccinated patients (15.4%) were not taking any immunomodulatory drug at the time of COVID-19; seven (53.8%) were on steroids, and four other patients (30.8%) were taking other immunomodulatory drugs. Regarding vaccinated patients, two (14.3%) were not taking any immunomodulatory drug at the time of infection, six (42.9%) were treated with steroids, five (35.7%) received monthly courses of IVIg alone or in combination with steroids/pyridostigmine, and the remaining four (28.6%) were on other immunomodulatory drugs (Table 1).

### 3.2. Course of SARS-CoV-2 Infection in Vaccinated and Non-Vaccinated Patients

The frequency of the severe course of COVID-19 was significantly greater in non-vaccinated patients compared to vaccinated cases (*p* = 0.004), as further confirmed by the significantly higher proportion of patients who were hospitalized (*p* = 0.004) and required non-invasive ventilation (*p* = 0.04) (Table 2). In our study population, only one non-vaccinated patient suffered from exacerbation of MG.

In vaccinated patients, the hospitalization, severe course, and mortality percentages were all 7.1%; in particular, only one patient, who was treated with rituximab, had a severe COVID-19 course, was hospitalized, and died (Table 2).

Three (21.4%) of the vaccinated patients underwent antiviral therapy with nirmatrelvir plus ritonavir with benefit, and neither of them had any adverse effects. However, in terms of SARS-CoV-2 infection treatment, the only significant difference between the two groups was the higher use of corticosteroids in non-vaccinated patients (*p* = 0.03, Table 2).

None of the vaccinated patients required oxygen therapy or ventilation, except for the single deceased patient, who was also intubated (Table 2).

Characteristics of deceased patients are reported in Table 3. Both the mean duration of MG in the deceased unvaccinated patients and the disease duration of the single deceased vaccinated patient were four years. None of the deceased patients had a history of thymoma or had undergone thymectomy.

### 3.3. Comparison of MG Features between Non-Vaccinated Deceased and Surviving Patients

**Figure 1:**
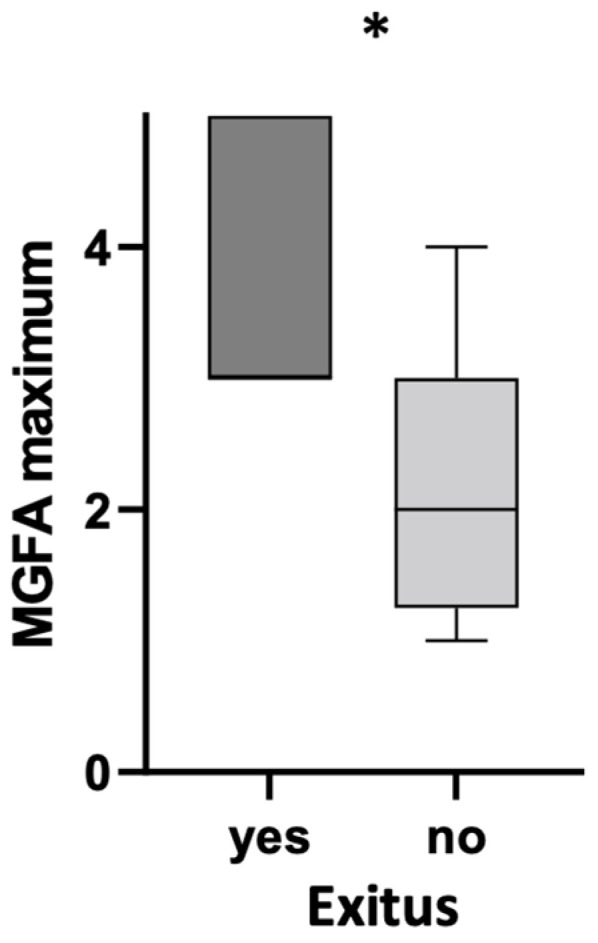
Boxplots showing maximum MGFA (Myasthenia Gravis Foundation of America) class that was reached in clinical history in non-vaccinated deceased and surviving patients. *: *p* < 0.05.

When we compared non-vaccinated deceased patients with survivors, we found that, although at the time of SARS-CoV-2 infection MG was stable and comparable between the two groups (median MGFA Class II), the median MGFA class that was reached in the past clinical history was significantly higher in deceased patients compared to survivors (*p* = 0.03) (Figure 1).

No other tested variables significantly differed between the two groups, including thymectomy, the number of past myasthenic exacerbations/crises, and past immunosuppressive therapy. 

### 3.4. Association between Clinical Features and the Course of COVID-19 in Vaccinated and Non-Vaccinated Patients

When we separately evaluated in non-vaccinated and vaccinated patients the association between clinical features and COVID-19 course, we observed that older age at MG onset, as well as at the time of infection, were significantly associated (Spearman r 0.63, *p* = 0.03 and Spearman r 0.60, *p* = 0.04, respectively) with a more severe course of COVID-19 in non-vaccinated patients (Table 4). Conversely, in the group of vaccinated patients, the only variable associated with a more severe course of COVID-19 was the Maximum MGFA Class reached in clinical history (Spearman r 0.57, *p* = 0.03). 

## 4. Discussion

This is a retrospective study evaluating all MG patients referring to five Ligurian hospitals, which cover almost the whole area of this region, so we think that the studied sample is well representative of the local myasthenic population. We evaluated patients with pre-existing gMG presenting with COVID-19 when attending the emergency department or routine consultation at the Neurologic Clinic, and we also collected data about MG patients evaluated via telemedicine, so we were able to evaluate even the mildest cases of SARS-CoV-2 infection.

The first part of the study focused on the first two pandemic waves to explore the impact of MG on COVID-19 course without the effect of vaccination and the new anti-viral therapies, which may have mitigated the consequences of the infection in all subjects, but particularly in frail ones, after November 2020. In the second part of the study, we further compared non-vaccinated patients to those subjected to anti-SARS-CoV-2 vaccination to evaluate the impact of vaccination on the gMG population.

The mortality rate that we observed in non-vaccinated patients was considerably high, in agreement with previous reports. Preliminary data from the COVID-19 Associated Risks and Effects in Myasthenia Gravis (CARE-MG) registry have demonstrated a mortality rate of 24% and a rate of exacerbation or myasthenic crises of 40% in 91 MG patients with COVID-19 [16]. A study conducted by Roy et al. has also shown that, while the rate of SARS-CoV-2 infection among myasthenic patients was comparable to the one observed in the general population, the risk of hospitalization and death was greater in MG cases [7].

Notably, in our study, we evaluated numerous parameters in order to disambiguate whether demographic factors (such as age and sex) and/or clinical features (including disease duration and the severity of MG) could predict a more severe course of COVID-19. As expected, older age both at the onset of MG and at the time of SARS-CoV-2 infection was associated with a more severe course. Interestingly, this association was observed only in non-vaccinated patients, probably thanks to the protective role of vaccination, which may have reduced the age-related risk. 

These findings, together with the observation of an association of greater MG severity with a more severe COVID-19 course, are in agreement with a recent study by Jakubíková and colleagues in which older age, long-term corticosteroid treatment, and poor control of MG (according to MGFA classification) prior to SARS-CoV-2 infection emerged as the most important predictors of more severe COVID-19 course [15]. The novelty of our study is that the association of older age both at the onset of MG and at the time of infection with a severe course of COVID-19 was observed only in non-vaccinated patients.

Yang et al. found that comorbid diseases, including cardiovascular diseases, represented important risk factors for severe COVID-19 outcomes [17], and a significant proportion of our patients had comorbidities, particularly cardiovascular ones, so this may have influenced the patients’ outcomes. The proportion of patients with BMI > 25 kg/m^2^ was higher among our unvaccinated patients, but the frequency of all comorbidities considered together was comparable between vaccinated and unvaccinated patients.

Interestingly, we found that the maximum MGFA class reached in clinical history was significantly higher in non-vaccinated patients who died than in survivors. All deceased patients, both vaccinated and unvaccinated, had relatively good compensation MG at the time of infection but had had particularly severe gMG in their past clinical history. A French multicentric, retrospective study on 3558 MG patients (34 of which had COVID-19) demonstrated that an MGFA Class ≥ IV at the time of SARS-CoV-2 infection was associated with a severe course of COVID-19 (the death rate in this population was 14.7%) [18]. Our result, although different from the one obtained in this large French cohort [18], is in line with the hypothesis that severe MG with previous myasthenic exacerbations/crises may be a risk factor for severe outcomes during COVID-19.

It is plausible to speculate that either patients with bulbar and respiratory forms might be more susceptible to respiratory failure when exposed to respiratory viruses, or that a severe autoimmune disease might trigger the COVID-19 Cytokine Storm [19], which, in turn, is associated with a more frequent fatal outcome. In this context, the observation of a less incisive impact of COVID-19 in other neuromuscular diseases with bulbar and respiratory muscle involvement, such as amyotrophic lateral sclerosis (ALS), seems to argue in favor of the second hypothesis [20,21].

Immunocompromised state related to immunotherapy may increase the susceptibility to infection, and this, in turn, is a well-recognized trigger of symptom exacerbation in patients with MG [2]. However, in our population, there were no significant differences regarding the type of therapy taken in the past or at the time of infection, and only one exacerbation of MG occurred in non-vaccinated patients. All our patients were in a stable disease phase when they contracted the infection; most of them were receiving pyridostigmine, low doses of steroids, or other immunomodulatory drugs. In our series, the treatment of patients who had a severe course did not differ from the treatment that was administered to mild cases, but the reduced numerosity of patients in our study does not allow us to argue in favor of the safety of immunomodulatory therapies during SARS-CoV-2 infection.

In our sample, myasthenic exacerbations or crises during the infection occurred only in one unvaccinated patient, while other studies have reported greater frequencies [13]. The administration of IVIg therapy resulted in a prompt amelioration, confirming the safety of this therapy in the case of myasthenic crises triggered by infections.

Vaccinated patients exhibited significantly better outcomes than non-vaccinated cases, but one vaccinated patient died because of pneumonia with respiratory failure requiring endotracheal intubation. This patient had gMG MuSK+ and was on rituximab therapy (an anti-CD20 monoclonal antibody), and this may have affected his response to vaccination [22,23,24]. In a recent meta-analysis, a significantly reduced humoral response was found in multiple sclerosis patients treated with anti-CD20 after receiving COVID-19 vaccination [11]. Despite the reduction in humoral responses, some studies have shown that stronger SARS-CoV-2 specific T cell responses determine a less severe infection [25], and in patients treated with anti-CD20 therapy, robust T cell responses to SARS-CoV-2 vaccination have been reported [26]; therefore, vaccination may confer protection even in the absence of an identifiable humoral response. Accordingly, Lupica and colleagues have provided data supporting the use of vaccines in myasthenic patients, even during active immunosuppressive and immunomodulatory treatments [10].

During the study period, several variants of SARS-CoV-2 followed one another (Alpha, Delta, and Omicron), with different contagiousness, pathogenicity, and severity of infection, so this could be a confounding factor in the evaluation of our patients. The Omicron variant seems to be less lethal but more contagious, and, due to the end of many previous COVID-19 restrictions, social contacts (and, therefore, the possibility of contagion) significantly increased during the last months of the study period. Doron and colleagues have previously reported mild to moderate symptoms both in the general population and in myasthenic patients during the omicron wave; however, revising mortality rates after the wave still demonstrated that MG patients are at higher mortality risk compared to the general population regardless of age [8], further supporting our results. 

The experience of physicians regarding COVID-19 has also changed over time, and this may have also influenced the patients’ outcomes observed in our study. It is indeed noteworthy that three of the vaccinated patients of our study also took the oral antiviral drug nirmatrelvir/ritonavir, which has been proven safe and effective in controlling the severity of the infection [27]. However, the only therapy administered more frequently in one of the two groups was the one with corticosteroids, which was more largely used in the unvaccinated group.

The present study is not without limitations. Despite having collected all the patients followed up in the main Ligurian hospitals, indeed, the examined sample size was moderately reduced, a factor possibly preventing a definitive interpretation of the obtained findings. The second shortcoming deals with the limited types of SARS-CoV-2 vaccines studied, since all patients included in the study received mRNA vaccines. However, it should be considered that mRNA vaccines are usually the ones preferred in myasthenic patients in order to prevent an increased risk of MG exacerbations associated with live attenuated vaccines. Thus, the data presented reflect the most frequent clinical management and can be considered to be representative of the vaccinated myasthenic population.

Despite these limitations, the present study confirms the particular fragility of MG patients, especially older ones and those with greater MG severity in the clinical history, and above all, highlights a protective role of anti-SARS-CoV-2 vaccination such as to reduce the age-related risk.

## 5. Conclusions

International and Italian guidelines have considered MG patients to be frail and prioritized their vaccination, as well as the vaccination of other patients affected by autoimmune diseases such as Multiple Sclerosis. Our data further support this view. The impact of SARS-CoV-2 infection was indeed considerably more negative in non-vaccinated cases, and only in this group, older age both at the onset of MG and at the time of infection correlated with a more severe course of COVID-19. Since 2021, the vaccination has been offered to the entire population with an important protective effect, and accordingly, the course of the infection in our cohort of vaccinated myasthenic patients has been found to be much less severe. However, it should be considered that there are conflicting data regarding the effect of any immunosuppressant drugs on the response to the anti-SARS-CoV-2 vaccine, so, in these patients, treatment with monoclonal antibodies or anti-viral drugs targeting SARS-CoV-2 should be started as early as they are tested positive, regardless of symptoms.

## Figures and Tables

**Table 1:** Demographic and MG features of patients both in clinical history and at the time of SARS-CoV-2 infection.

Variables	Vaccinated (n)		*p* Value
	No (13)	Yes (14)	
Sex M, n (%) F, n (%)	6 (46.2%) 7 (53.8%)	3 (21.4%) 11 (78.6%)	0.23
Age, years	68 ± 13	63 ± 17	0.56
Age at MG onset, years	61 ± 19	55 ± 19	0.62
Age at the time of COVID-19, years	67 ± 13	63 ± 18	0.78
Duration of disease, years	6.7 ± 9.5	7.3 ± 6.9	0.37
BMI, n (%) <25 >25	6 (46.1%) 7 (53.9%)	12 (85.7%) 2 (14.3%)	**0.04**
Antibody status, n (%) AchR+ MuSK+ Seronegative	13 (100%) 0 0	12 (85.7%) 2 (14.3%) 0	0.48 0.48 -
History of thymectomy, n (%) Yes No	4 (30.8%) 9 (69.2%)	7 (50%) 7 (50%)	0.44
Maximum MGFA Class in clinical history, n (%) <3 ≥3	5 (38.5%) 8 (61.6%)	1 (7.1%) 13 (92.9%)	0.07
MGFA Class at the time of COVID-19, n (%) <3 ≥3	11 (84.6%) 2 (15.4%)	9 (64.3%) 5 (35.7%)	0.38
Past myasthenic exacerbations, n (%) 0 ≥1	8 (61.5%) 5 (38.5%)	6 (42.9%) 8 (57.1%)	0.44
Comorbidities, n (%) No 1 ≥2	5 (38.5%) 5 (38.5%) 3 (23.0%)	4 (28.6%) 3 (21.4%) 7 (50%)	0.69 0.41 0.23
Treatment of MG at the time of COVID-19 None Pyridostigmine alone Corticosteroids Other immunomodulatory drugs Monthly IVIg	1 (7.7%) 1 (7.7%) 7 (53.8%) 4 (30.8%) 2 (15.4%)	1 (7.1%) 1 (7.1%) 6 (42.9%) 4 (28.6%) 5 (35.7%)	1.00 1.00 0.70 1.00 0.38

BMI: body mass index; M: males; F: females; MG: myasthenia gravis; COVID-19: coronavirus disease 2019; AChR+: anti-acetylcholine receptor antibody positive; MuSK+: anti-muscle-specific tyrosine kinase positive; MGFA: Myasthenia Gravis Foundation of America; IVIg: intravenous immunoglobulin. *p* values refer to Mann–Whitney *U*-test and Fisher exact test.

**Table 2:** The course of SARS-CoV-2 infection and the need for oxygen and ventilatory support in the 14 vaccinated and 13 non-vaccinated myasthenic patients.

Variables	Vaccinated (n)		*p* Value
	No (13)	Yes (14)	
Hospitalization, n (%) Yes No	8 (61.5%) 5 (38.5%)	1 (7.1%) 13 (92.9%)	**0.004**
Hospitalization days (for hospitalized patients)	14 ± 10	30	-
Severe COVID-19, n (%) Yes No	8 (61.5%) 5 (38.5%)	1 (7.1%) 13 (92.9%)	**0.004**
MG crises/exacerbations during COVID-19, n (%) Yes No	1 (7.7%) 12 (92.3%)	2 (14.3%) 12 (85.7%)	1.00
Treatment for COVID-19, n (%) None or symptomatic treatment Remdesivir Nirmatrelvir+ritonavir Antibiotics Steroids Hydroxychloroquine Anakinra	6 (46.2%) 2 (15.4%) 0 5 (38.5%) 6 (46.2%) 1 (7.7%) 1 (7.7%)	9 (64.3%) 0 3 (21.4%) 1 (7.1%) 1 (7.1%) 0 0	0.44 0.22 0.22 0.07 **0.03** 0.48 0.48
IVIg therapy during SARS-CoV-2 infection, n (%) No Yes	11 (84.6%) 2 (15.4%)	14 (100%) 0	0.22
Oxygen therapy/ventilatory support, n (%) No Oxygen therapy NIV ETI	5 (38.5%) 3 (23.1%) 4 (30.8%) 1 (7.7%)	13 (92.9%) 0 0 1 (7.1%)	**0.004** 0.09 **0.04** 1.00
Exitus, n (%) Yes No	4 (30.8%) 9 (69.2%)	1 (7.1%) 13 (92.9%)	0.16

COVID-19: coronavirus disease 2019; MG: myasthenia gravis; IVIg: intravenous immunoglobulin; NIV: non-invasive ventilation; ETI: endotracheal intubation. *p* values refer to Fisher exact test.

**Table 3:** Clinical characteristics of deceased patients.

Variable	Patient 1	Patient 2	Patient 3	Patient 4	Patient 5
Age, years	68	60	61	87	72
Sex	M	M	M	F	M
Vaccination against SARS-CoV-2	No	No	No	No	Yes
Duration of MG, years	3	8	4	2	4
Antibody status	AchR+, titin+	AchR+	AchR+, titin+, ryanodine+	AchR+	MuSK+
Thymectomy	No	No	No	No	No
Thymoma	No	No	No	No	No
Maximum MGFA in clinical history	V	IIIB	V	IIIA	V
MGFA at the time of infection	I	II	I	IIIA	II
MG therapy at the time of infection	Prednisone 2.5 mg, pyridostigmine	Prednisone 37.5 mg, pyridostigmine	Mycophenolate mofetil	Prednisone 20 mg, pyridostigmine	Rituximab
Comorbidities	Overweight	Ischemic heart disease, hypertension, T2D, CKD, overweight	Hypertension, overweight	Ischemic heart disease, AF, hypertension	T2D
Pneumonia/Sepsis	No	Sepsis	No	Pneumonia	Pneumonia and sepsis
Hospitalization days	2	15	4	13	30
MG exacerbation/crisis	No	No	No	No	Yes
Treatment	IVIg, steroids, oxygen therapy	Remdesivir, antibiotics, LMWH, ETI	NIV	Hydroxychloroquine, steroids, antibiotics, anakinra, LMWH, NIV	Antibiotics, ETI

M: males; F: females; SARS-CoV-2: severe acute respiratory syndrome-coronavirus-2; MG: myasthenia gravis; AChR+: anti-acetylcholine receptor antibody positive; MuSK+: anti-muscle-specific tyrosine kinase positive; MGFA: Myasthenia Gravis Foundation of America; T2D: type two diabetes mellitus; CKD: chronic kidney disease; AF: atrial fibrillation; IVIg: intravenous immunoglobulin; LMWH: low-molecular-weight heparin; NIV: non-invasive ventilation; ETI: endotracheal intubation.

**Table 4:** Correlation analyses between clinical features and the course of COVID-19 in vaccinated and non-vaccinated patients.

Variables	Non-Vaccinated Patients (13)		Vaccinated Patients (14)	
	r	*p* Value	r	*p* Value
Age at MG onset	0.63	**0.03**	0.06	0.82
Age at the time of COVID-19	0.60	**0.04**	0.04	0.89
Duration of disease	−0.23	0.44	−0.09	0.74
Maximum MGFA Class in clinical history	0.44	0.12	0.57	**0.03**
MGFA Class at the time of COVID-19	0.30	0.32	0.24	0.39

MG: myasthenia gravis; COVID-19: coronavirus disease 2019; MGFA: Myasthenia Gravis Foundation of America. Values are given as Spearman correlation coefficients (r) and *p* values.

## Data Availability

Not applicable.
